# Delayed Onset Sciatic Nerve Palsy Secondary to Wound Hematoma following Anticoagulant Therapy Post-Bipolar Hemiarthroplasty - an Uncommon Complication: A Case Report

**DOI:** 10.5704/MOJ.1907.010

**Published:** 2019-07

**Authors:** G Balaji, Y Sriharsha, D Sharma

**Affiliations:** Department of Orthopaedics, Jawaharlal Institute of Postgraduate Medical Education and Research (JIPMER), Pondicherry, India

**Keywords:** sciatic nerve, sciatic neuropathy, venous thrombosis

## Abstract

A 58-year old female patient presented to us with a three months’ old fracture of the neck of femur. She underwent bipolar hemiarthroplasty. In the immediate postoperative period, she developed deep vein thrombosis for which she was started on anticoagulant therapy. Patient had persistent discharge from the wound since then and underwent regular dressings. On the eighth post-op day, she developed sciatic nerve palsy secondary to wound haematoma. The haematoma was decompressed immediately and she had a dramatic improvement in pain but her neurological deficit persisted. The wound healed completely without any complications. At three months follow up, she had recovered completely with grade 5/5 power in ankle and foot and full sensory recovery in the sciatic nerve distribution. She was ambulating comfortably with a walker. At final follow up around 20 months post-operation, she was pain-free and walking without any support. The wound had healed completely.

## Introduction

Sciatic nerve palsy is a rare but potentially disabling complication in hip arthroplasty. There are few isolated reports of sciatic nerve palsy due to hematoma formation and it remains a rare complication1^-5^. We describe a case of delayed sciatic nerve palsy due to hematoma formation. Informed consent was obtained from the patient regarding case data to be used for research and educational purposes.

## Case Report

**Fig. 1:**
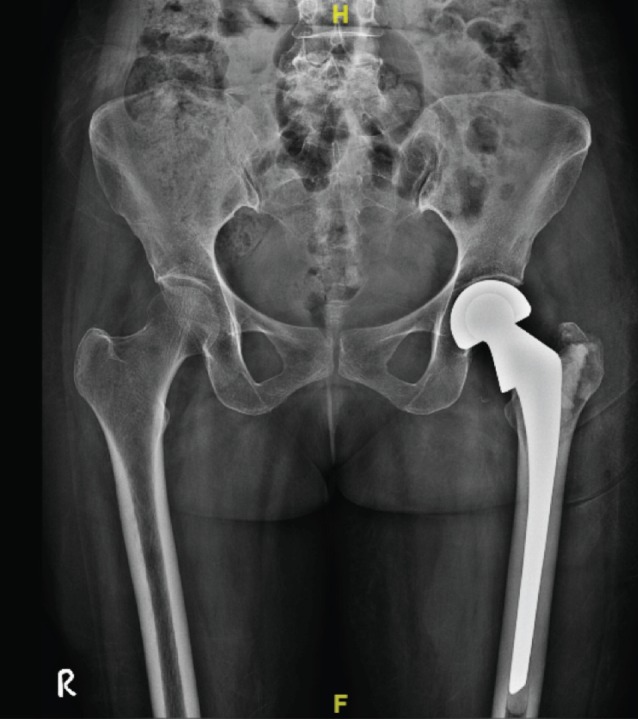
Plain AP radiograph of the pelvis in the immediate postoperative period with bipolar prosthesis in situ.

A 58-year old female patient presented to us with pain in the left groin and inability to weight bear for three months following a fall from stairs at her home. She had taken traditional treatment in the form of oil massage and splinting initially. The patient was a known case of seropositive rheumatoid arthritis on regular disease-modifying drugs. She was diagnosed to have a sub-capital neck of femur fracture. She underwent cemented bipolar hemiarthroplasty through posterior (Moore’s) approach using Ormed implants (Batch No. 150925/2, Lot no. BP 037) with 41mm head size and Depuy Gentamycin bone cement (Fig. 1). Intra-operatively there were no complications observed and surgical wound was closed in layers with suction drain. Eight hours later, the patient had completely recovered from spinal anaesthesia with no sensory or motor deficit on examination.

On the first post-operative day, the patient was taught isometric quadriceps exercises along with ankle range of motion exercises. On the second day, the drain was removed and she was mobilised weight bearing as tolerated with a walker. She developed swelling and erythema of the leg along with calf tenderness on the third day. Duplex ultrasonogram of the left lower limb revealed deep vein thrombosis (DVT) of the femoral vein (partial thrombus occluding the lumen and non-compressible with probe). She was started on Inj. Enoxaparin 40mg SC BD and Tab. Warfarin 5mg OD. Two days post-anticoagulation therapy, the patient developed severe pain in the left gluteal region with increasing intensity radiating to the great toe. A fluctuant swelling was noted over the operated site with associated serosanguinous discharge. Few skin staples were removed and around 100ml of fresh hematoma was drained from the wound, following which the patient was comfortable and symptoms reduced. On the eighth postoperative day, the patient again developed increased pain in the gluteal region radiating to the great toe associated with numbness of the foot. Examination revealed foot drop along with weakness of knee flexion and loss to fine touch and pinprick over dorsum and plantar aspect of the foot suggestive of sciatic palsy. Ultrasonogram (USG) showed a collection of fluid at the sub-muscular plane of the hip and around the sciatic nerve compressing it and a persisting thrombus in the femoral vein with no change as compared to the previous scan. The patient underwent an emergency wound debridement and decompression of the sciatic nerve.

**Fig. 2:**
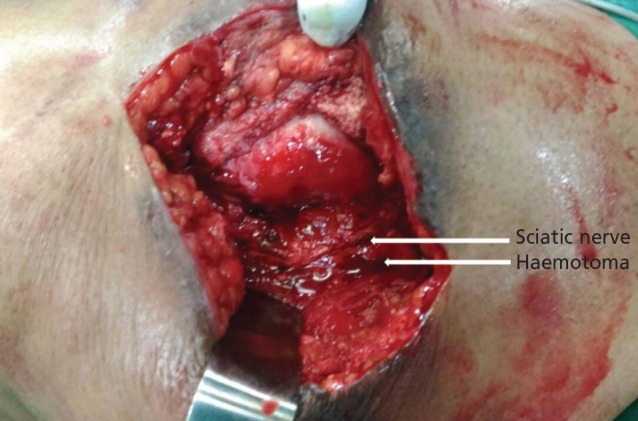
Intraoperative image showing haematoma around the sciatic nerve.

**Fig. 3:**
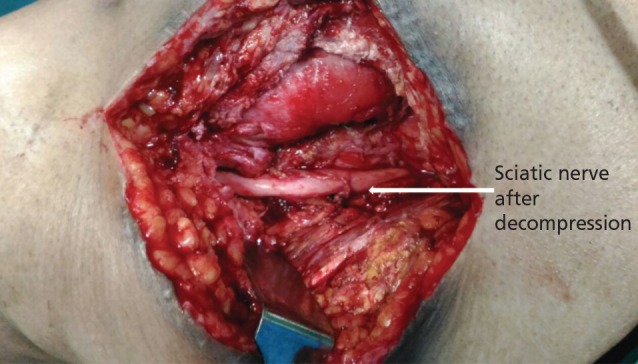
Image showing complete decompression of sciatic nerve.

Intra-operatively, there was seropurulent collection in the submuscular plane compressing the sciatic nerve (Fig. 2,3). The joint was dislocated and thorough wound debridement carried out. There was no fluid collection in the joint. The prosthesis was stable. The nerve was decompressed completely and the joint was relocated. The wound was closed in layers with a drain *in situ*. The fluid and tissues were sent for culture and sensitivity study.

The culture revealed methicillin-resistant *Staphylococcus aureus*. She was started on Inj. Vancomycin 15mg/kg TDS for two weeks. She had dramatic relief from her pain post-decompression but her neurological deficits persisted. The wound healed completely without any complications. In the meantime, anticoagulant therapy was titrated and she was started on Tab. Warfarin 5mg daily. Staples were removed on the sixteenth post-operative day. She was ambulated with a walker, weight bearing as tolerated, and was discharged on oral Linezolid 600mg twice a day for four weeks.

At three months follow up, her sciatic nerve had recovered completely with grade 5/5 power in ankle and foot and full sensory recovery in the sciatic nerve distribution. She was ambulating comfortably with a walker. She was advised to continue anticoagulant therapy for another three months by the physician. At the end of 20 months follow-up, the wound had healed completely, she was pain-free and walking without any support.

## Discussion

Sciatic nerve palsy following hip arthroplasty is a well known complication secondary to direct injury to the nerve, traction injury to the nerve due to over lengthening of the limb (particularly in neglected cases and developmental dysplasia of the hip), compression due to retractors, damage to the nerve due to thermal injury from cement or fraying of the nerve over cement osteophyte^4,5^. Delayed sciatic nerve palsy secondary to wound haematoma in the postoperative period is a rare phenomenon^1,2^. Very few cases have been reported in the literature on delayed sciatic palsy secondary to wound haematoma. Fleming *et al*^3^ were among the earliest to report five cases of sciatic nerve palsy secondary to bleeding after hip surgery. Sorenson *et al*^2^ reported two cases of wound haematoma-induced sciatic nerve palsy following hip arthroplasty. They hypothesised that increase incompartment pressure beneath the closed fascia was responsible for nerve palsy.

Butt *et al*^4^ reported six cases of wound haematoma-induced sciatic nerve palsy post-THA secondary to anticoagulant therapy. They also found that five of their patients were less than 70kg in weight and received a full prophylactic dose of anticoagulants which could be the cause for haematoma formation. Hence they advised a reduced dose of anticoagulants in such patients.

There is no consensus regarding time from onset of sciatic nerve irritation to decompression and final clinical outcome in terms of return of sciatic nerve function^5^.

Sorenson *et al*^2^ in their report decompressed after 12 hours of initial complaints in one patient and after six hours in the other. The first one did not show nerve recovery while the second patient had good recovery of nerve function. They suggested that the timing of decompression plays a key role in the recovery of nerve function.

Austin *et al*^1^ reported a case of late sciatic nerve palsy following THA on the 18th postoperative day secondary to wound haematoma. Though they decompressed immediately, their patient had persistent foot drop till final follow up. Beksac *et al*^5^ reported a case which was decompressed 26 hours after diagnosis. At nine months follow up, the patient still had a persistent neurological deficit. Our patient was decompressed within 12 hours after onset of foot drop. She showed good improvement with full recovery of sciatic nerve function.

If a patient required post-operative thromboprophylaxis due to high risk of thromboembolism, the clinician should be cautious regarding the bleeding complications that might occur. The dose of anticoagulant should be decided as per the weight of the patient^4^. Our patient had wound haematoma which was clinically evident as a swelling over the groin and serosanguinous discharge from the wound. Also ultrasonogram of the hip showed signs of collection compressing the sciatic nerve. Once diagnosis of wound hematoma is confirmed, immediate decompression is recommended.

In conclusion, sciatic nerve palsy is a significant disabling complication. Prompt diagnosis and timely intervention play a key role in reducing the overall complication rate and the serious morbidity associated with it.
